# Hereditary Hemorrhagic Telangiectasia in a Sudanese Patient

**DOI:** 10.1155/2020/6395629

**Published:** 2020-12-14

**Authors:** Omer Ali Mohamed Ahmed Elawad, Ahmed Abdalazim Dafallah Albashir, Mohammed Mahgoub Mirghani Ahmed, Ahmed Ali Mohamed Ahmed Elawad, Osman Eltieb Elbasheer Mohamed

**Affiliations:** ^1^Wad Medani Teaching Hospital, Wad Medani, Sudan; ^2^Faculty of Medicine, University of Gezira, Wad Medani, Sudan

## Abstract

**Background:**

Hereditary hemorrhagic telangiectasia (HHT) also known as Osler–Weber–Rendu syndrome is a rare autosomal dominant disorder, which results in vascular dysplasia affecting mainly visceral and mucocutaneous organs. *Case Presentation*. A 65-year-old woman with a 12-year history of recurrent spontaneous epistaxis presented with shortness of breath, easy fatigability, and bilateral lower limb edema. Her family history was significant for definite hereditary hemorrhagic telangiectasia in first-degree relatives. During the previous 15 days, she has experienced three episodes of recurrent nasal bleeding. She has a background of chronic mitral regurgitation. Physical examination revealed telangiectases in her tongue, lower lip, and hand in addition to signs of congestive heart failure. The patient met 3\4 Curacao criteria and had a definite HHT. Her laboratory workup revealed a hemoglobin count of 5.4 g/dl. Echocardiography revealed a left systolic ejection fraction of 51% with left atrial dilatation and severe mitral regurgitation. Chest X-ray showed features of cardiomegaly and pulmonary edema. The abdominal ultrasonography showed enlarged liver size with homogenous texture and congested hepatic veins without features of hepatic AVMs. She was treated with intravenous frusemide, iron supplement, tranexamic acid, blood transfusion, and nasal packing.

**Conclusions:**

HTT usually passes unnoticed in Sudan. The rarity of HHT, difficulties in affording diagnostic imaging studies, and low clinical suspicion among doctors are important contributing factors. Anemia resulting from recurrent epistaxis might have an influential role in precipitating acute heart failure in those with chronic rheumatic valvular disease.

## 1. Introduction

Hereditary hemorrhagic telangiectasia (HHT) is a rare genetic disorder, which has an autosomal dominant pattern of inheritance. The estimated prevalence is 1/5000–1/8000 of population [1]. It is characterized by diffuse telangiectases (e.g., ruby-coloured papules that blanch with pressure), recurrent epistaxis, and widespread arteriovenous malformations (AVMs). Males and females are equally affected.

The diagnosis is based on the presence of three out of four criteria, called Curaçao criteria, which include recurrent spontaneous epistaxis, small systemic telangiectasia (due to AVMs), visceral involvement, and a strong family history of HHT (Table 1).

Although the symptoms usually vary according to age, epistaxis is usually the commonest clinical feature [2].

To the best of our knowledge, this is the first reported case of HHT in Sudan. The rarity of the disease, difficulties in affording the diagnostic imagining and genetic testing, and low clinical suspicion among doctors, all of which pose a challenge in the diagnosis and management of HHT, particularly in resources- limited hospitals.

## 2. Case presentation

A 65-year-old Sudanese woman came to a university hospital, Sudan, complaining of shortness of breath (New York Heart Association (NYHA) class 3), easy fatigability, and bilateral lower limb edema. The patient had no history of diabetes mellitus or hypertension. During the previous 15 days, she has experienced three episodes of recurrent nasal bleeding, which were not related to any particular activity, posture, or seasonal variation. There was no bleeding from other orifices. For many years, episodes of nasal bleeding occurred frequently, and the patient received medical advice at their rural hospital. During those episodes, the patient underwent anterior nasal packing, transfused, and then discharged on iron supplements. The patient then came to a university hospital for more advanced evaluation. During the patient workup, the patient experienced an episode of epistaxis for which she underwent anterior nasal packing to control the epistaxis and received tranexamic acid 500 mg tabs TDS, topical tranexamic acid, and packed cells for transfusion. The workup revealed that the patient is a known case of chronic mitral regurgitation, and she was planned for mitral valve replacement, which was not performed due to financial challenges.

The family history was significant for definite HHT in the form of recurrent spontaneous nose bleeding and the same small erythematous lesions (telangiectases) in her grandmother, her son, and her daughter.

**Figure 1:**
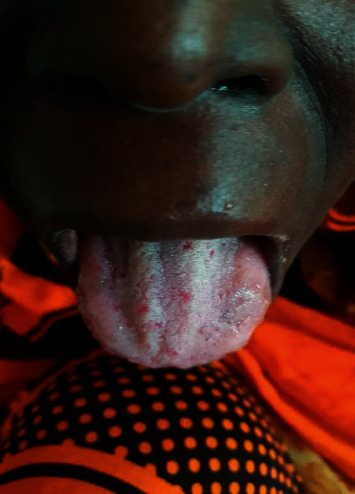
The tongue showing multiple small telangiectasias.

**Figure 2:**
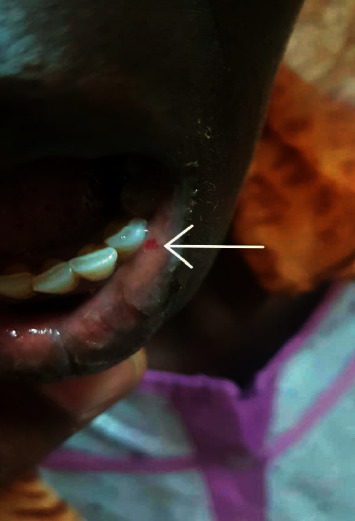
Small telangiectasia at the left border of the lower lip.

**Figure 3:**
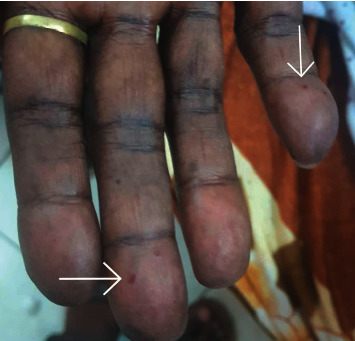
Telangiectasias at the palmar aspect of the distal phalanx of the middle finger and the distal phalanx of the little finger.

Physical examination revealed that the patient looked ill, pale, and not jaundiced. JVP was raised. Her BP was 110/60, pulse rate was 88 BPM, and it was not collapsing. The oxygen saturation was 90% on room air. Mouth examination revealed multiple small erythematous lesions, which blanch on pressure (telangiectases) on her tongue (Figure 1) and one lesion on her lower lip (Figure 2). Hand examination revealed one telangiectasia at the palmar aspect of her right hand (Figure 3). Her cardiovascular examination revealed a pan systolic murmur at the apical area, which radiated to the axilla, and bilateral fine basal crepitations. The liver was tender and palpable 8 cm below the right costal margin without audible bruit, and there is bilateral lower limb edema.

The laboratory investigations at admission revealed hypochromic microcytic anemia (hemoglobin 5.4 g/dl). Other laboratory investigations are described in Table 2.

**Figure 4:**
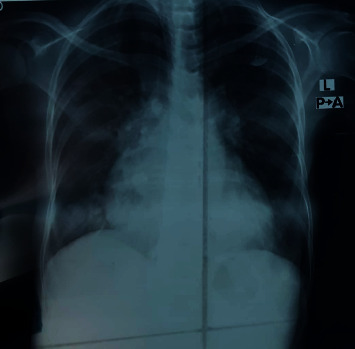
Chest X-ray posterior/anterior view revealed upper lobe diversion and congested hilar shadows and patchy consolidation in lower lobes (features of pulmonary edema and cardiomegaly).

**Figure 5:**
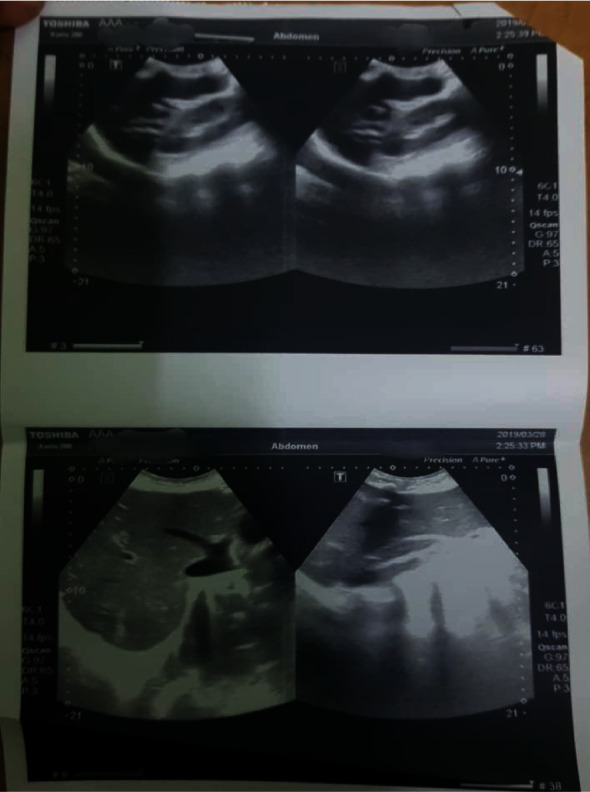
Abdominal US showing the liver enlarged in size (19 cm) with homogenous texture and congested hepatic veins.

ECG was noncontributory apart from sinus tachycardia. Chest X-ray revealed upper lobe diversion, congested hilar shadows and patchy consolidation in lower lobes (features of pulmonary edema and cardiomegaly) (Figure 4). Echocardiography showed a left systolic ejection fraction of 51% with left atrial dilatation and severe mitral regurgitation. The abdominal ultrasonography (US) displayed enlarged liver size (19 cm) with homogenous texture and congested hepatic veins. Hepatic arteriovenous malformations were not detected on ultrasonography (Figure 5).

Diagnostic challenges: CT pulmonary angiography was not done due to financial challenges. Contrast echocardiography is an alternative. It was intended to screen the patient for the presence of pulmonary AVMs. Screening can be performed with contrast echocardiography and then a CT-scan in case of a right-to-left shunt. The most feared complication of a pulmonary AVM, cq a right-to-left shunt, is brain abscess, due to paradoxical septical emboli, which are normally trapped in the pulmonary capillaries, but can pass through the pulmonary AVM. As long as the presence of a right-to-left shunt is not excluded in HHT patients, prophylactic antibiotics should be prescribed before any nonsterile procedures, to prevent brain abscess.

Contrast-enhanced CT abdomen was also not done due to financial challenges. It was meant to detect hepatic AVMs and to exclude high-output heart failure. However, clinically, there were no features of hyperdynamic circulation in the patient (e.g., collapsing pulse).

The diagnosis was definite HHT as she fulfilled three items of the Curacao criteria (spontaneous, recurrent nose bleeding, family history in a first-degree relative, and multiple telangiectases at characteristic sites such as tongue, lips, and hands). Anemia, which was attributed to recurrent epistaxis and nutritional factors, is thought to play an influential role in precipitating the heart failure in this patient with chronic rheumatic mitral regurgitation.

The patient was treated with intravenous frusemide, iron supplement, tranexamic acid, blood transfusion, and nasal packing. She was discharged on day 7 on lisinopril tablets and iron supplements in addition to counselling about home-preventive measures of epistaxis.

## 3. Discussion

Hereditary hemorrhagic telangiectasia (HHT) is a rare autosomal dominant disorder characterized by arteriovenous malformations (AVM) of the internal organs and mucocutaneous telangiectasias. These AVMs lack the intervening capillaries resulting in direct connections between arteries and veins. They can involve the skin, mucous membranes, brain, lung, gastrointestinal tract, and/or liver [3]. The most common clinical features are recurrent spontaneous nosebleeds which present in 50% of patients with HHT at the age of ten, with incidence increasing with age.

HHT was first described in 1864 in a man with a vascular malformation and recurrent epistaxis [4]. In 1996, Rendu first noticed the correlation between hereditary epistaxis and telangiectasia in a 52-year-old man [5]. Osler in 1901 and Weber in 1907 then identified the association between hemorrhagic lesions in the skin and mucous membranes and its familial inheritance [6, 7]. The name ‘hereditary hemorrhagic telangiectasia' described by Hanes in 1909 defines the characteristics of the disease [8].

Mutation in genes, including ACVRL1, ENG, and SMAD4 genes, are responsible for hereditary hemorrhagic telangiectasia. The ENG mutation can lead to hereditary hemorrhagic telangiectasia type 1, while type 2 hereditary hemorrhagic telangiectasia is caused by mutations in the ACVRL1 gene. Juvenile polyposis/hereditary hemorrhagic telangiectasia syndrome is caused by a mutation in the SMAD4 gene. It has been proposed that certain mutated HHT genes are associated with specific clinical features, for example, ACVRL1 mutation was found to be associated more with liver AVMs, spinal AVMs, epistaxis, and pulmonary hypertension. ENG mutations were seen to be associated more with pulmonary and brain AVMs, and MADH4 mutations were observed to be associated with juvenile colonic polyposis.

The diagnosis of HHT depends on diagnostic criteria called Curacao criteria (Table 1). Our patient was diagnosed as definite HHT as she fulfilled three items of the Curacao criteria (spontaneous, recurrent nose bleeding, family history in a first-degree relative, and multiple telangiectasias at characteristic sites such as tongue, lips, and hands).

In patients with HHT, heart failure usually occurs due to hepatic arterial-venous malformation, which causes high-output heart failure. In our patient, anemia, which was attributed to recurrent epistaxis and nutritional factors, is thought to play an influential role in precipitating the heart failure in this patient with chronic rheumatic mitral regurgitation. Iron deficiency anemia is a common complication of HHT; chronic blood loss from nasal mucosa or intestinal tract can result in depletion of iron storage, which in turn can lead to microcytic or normocytic anemia.

As per ESC Guidelines HF 2016, in the initial assessment of a patient with newly diagnosed heart failure, ferritin and transferrin saturation (TSAT) are the recommended diagnostic tests. The treatment is recommended when ferritin is <100 *µ*g/L, or ferritin is between 100 and 299 *µ*g/L and TSAT <20%.

There is no curative treatment for HHT; treatment is symptomatic which focuses on the predominant clinical feature and its severity. The first step in the management of epistaxis is patient counselling about the use of home-preventive measures such as nasal humidification, saline sprays, or ointment to avoid dryness of nasal mucosa and to decrease the risk of nasal trauma in the form of nasal picking and nasal blowing [9]. Other options included nasal packing, photocoagulation, intravascular embolization, and surgery. These methods have a high rate of recurrence with limited efficacy, and they are not available in many resources- limited hospitals. Drugs such as bevacizumab (an antivascular endothelial growth factor monoclonal antibody) are widely used in the treatment of high-output heart cardiac failure in HHT with adequate safety and efficacy [10]. Tamoxifen (antiestrogen medication) [11] and thalidomide (an antiangiogenic and immunomodulatory agent) [12] had been also investigated in the treatment of HHT.

According to ESC, treatment of heart failure with preserved ejection fraction (HFpEF) is the treatment of the underlying cause such as anemia; diuretics are recommended in congested patients with HFpEF to alleviate symptoms and signs.

Patients with HHT have a lower life expectancy than the general population; the prognosis depends on the severity of symptoms. The most common cause of death in HHT is heart failure and sepsis [13].

## 4. Conclusion

In conclusion, cases of HHT may have been passed unnoticed due to rarity of the disease that leads to low clinical suspicion among doctors. Other contributing factors include variation in clinical presentations of the disease, difficulties in affording the diagnostic imaging studies, and the imaging studies might not be available in many resourses-limited hospitals. Anemia resulting from the recurrent epistaxis might have an influential role in precipitating acute heart failure in those with chronic rheumatic valvular disease.

## Figures and Tables

**Table 1:** The Curacao diagnostic criteria for HHT.

Criteria	Characteristic
(1) Epistaxis	Recurrent and spontaneous nosebleeds (epistaxis), which may be mild to severe
(2) Telangiectases	Telangiectases are small red spots that disappear when pushed on, and they are found at characteristic sites (lips, oral cavity, fingers, and nose)
(3) Visceral lesions	For example, gastrointestinal telangiectasia (with or without bleeding), pulmonary arteriovenous malformations (AVM), hepatic AVM, cerebral AVM, and spinal AVM
(4) Family history	That is, first-degree relative such as brother, sister, parent, or child who meets these same criteria for definite HHT or has been genetically diagnosed.

If they meet 3 or more of the criteria, they are said to have a definite HHT. If patients meet 2, then they are said to have a possible diagnosis of HHT, and “unlikely” if 0 or 1 criterion is present.

**Table 2:** It describes the results of the blood investigations obtained for the patient.

Laboratory investigation	Value
Hb% MCV MCH TWBCs platelets	5.4 g/dl (13–17) 70 fl (80–96) 18.8 pg (26–34) 4,500/mm^3^ (4.0–10 × 103) 258,000/mm^3^ (150–400)
Blood urea	55 mg/dl (15–45)
Serum creatinine	1.5 mg/dl (0.6–1.3)
Random blood sugar	150 mg/100 ml(90–140)
HbA1c	5.3 % (up to 5.7%)
INR	1.4 (up to 1.5)
PT	13 s (12–14)
aPTT	25 s (35–45)
Ferritin	4.7 ng/ml(13–150)
Serum iron	29 ng/ml (50–150)

## Data Availability

The data used to support the findings of this study are included within the article.

## Abbreviations

HHT: Hereditary hemorrhagic telangiectasia
OWRD: Osler-Weber-Rendu disease
AVMs: Arteriovenous malformations
NYHA: New York Heart Association
BNP: Brain natriuretic peptide
HFpEF: Heart failure with preserved ejection fraction
ACVRL1: Activin A receptor-like type 1
ENG: Endoglin
TSAT: Transferrin saturation.
